# Supplementary material for: A high-resolution mRNA expression time course of embryonic development in zebrafish
Source: eLife. 2017 Nov 16;6:e30860. doi: 10.7554/eLife.30860 (PMC5690287; doi:10.7554/eLife.30860)
Supplement: Supplementary file 6. [file elife-30860-supp6.zip › biolayout-clusters-files/Cluster242-genes.html]

Cluster242


# Cluster242: Genes

| Ensembl ID | Gene Name | Chr | Start | End | Biotype |
| --- | --- | --- | --- | --- | --- |
| ENSDARG00000104457 | MPV17L | 3 | 36468227 | 36471019 | protein\_coding |
| ENSDARG00000095767 | dio3b | 20 | 54013075 | 54021192 | processed\_transcript |
| ENSDARG00000074396 | fscn2b | 12 | 34337214 | 34358538 | protein\_coding |
| ENSDARG00000057209 | plppr5a | 2 | 20604507 | 20665929 | protein\_coding |
| ENSDARG00000008306 | rdh5 | 22 | 10083191 | 10092198 | protein\_coding |
| ENSDARG00000017367 | rhbdf1b | 12 | 20105357 | 20181564 | protein\_coding |
